# Prevalence of *Alaria alata* mesocercariae in wild boars from Brandenburg, Germany

**DOI:** 10.1007/s00436-021-07178-9

**Published:** 2021-05-08

**Authors:** Carolyn Kästner, Nadja Seyhan Bier, Anne Mayer-Scholl, Karsten Nöckler, Martin Heinrich Richter, Annette Johne

**Affiliations:** Department for Biological Safety, German Federal Institute for Risk Assessment (BfR), Diedersdorfer Weg 1, 12277 Berlin, Germany

**Keywords:** *Alaria alata*, Prevalence, Wild boars, Germany, Epidemiology

## Abstract

Since 2002, *Alaria (A.) alata* mesocercariae (AM) have been found during routine *Trichinella* inspection of wild boars in many European countries. To date, human infection with AM through consumption of undercooked or raw AM infested wild boar meat cannot be excluded. In Germany, data on the parasite’s prevalence in wild boars are scarce. To better understand temporal and spatial fluctuations of this parasite, this study investigated the prevalence of AM in wild boars in the German federal state of Brandenburg during three hunting seasons from 2017 to 2020. In total, 28.3% (100/354, 95% CI: 23.3–33.3%) of all wild boars sampled in eight counties of Brandenburg were tested positive for AM by *Alaria alata* mesocercariae migration technique (AMT). AM were detected in wild boars from seven different counties. Samples from one county (Havelland) tested completely negative for AM (0/16). Prevalences of the seven AM positive counties of Brandenburg ranged from 11.5 (3/26, 95% CI: 2.5–30.1%) in Märkisch-Oderland to 64.1% (25/39, 95% CI: 47.2–78.8%) in Uckermark. An association between sex and *A. alata* positivity could not be determined. A statistically significant increase in frequency of older AM positive wild boars was observed (*p* = 0.001). For a nationwide assessment of the prevalence of *A. alata* in wild boars and the risk for consumers of ingesting viable AM by consumption of raw or undercooked AM infested wild boar meat, further long-term studies in different regions of Germany are needed.

## Introduction

Wild boar meat infested with foodborne parasites can lead to human infection if consumed raw or undercooked (Ruiz-Fons 2017; EFSA 2018). One of these parasites, *Alaria (A.) alata* mesocercariae (AM), are a larval stage of the trematode *A. alata* which have been detected during routine *Trichinella* examination of wild boars in many European countries since 2002 (Möhl et al. 2009; BfR 2017).

*A. alata* is a parasite which has a complex three-host life cycle. The adult trematodes live in the intestine of several carnivores which serve as definitive hosts (Odening 1961). These adult worms excrete eggs which hatch in water and release miracidiae. These develop further in a water snail host, e.g. *Planorbis* species (Odening 1961; Hiepe 1985). In this host, the miracidiae reproduce and mature for about 1 year (reviewed by Möhl et al. (2009)) before they actively leave the water snail as cercariae and enter the second intermediate hosts in the water. In these second intermediate hosts (e.g. adult frogs, tadpoles or other amphibia), the cercariae develop into mesocercariae (Odening 1961). These AM infested second intermediate hosts can further be ingested by definitive as well as paratenic hosts (Odening 1961). In case of *A. alata*, amphibians, reptiles, birds and mammals such as wild boars, minks, badgers as well as potentially humans rank among paratenic hosts in the developmental cycle (Odening 1961; Deplazes et al. 2012; Takeuchi-Storm et al. 2015; Rentería-Solís et al. 2018). In these hosts, larval trematodes go through the intestinal wall into the muscles of the anterior body region and/or settle down in or on several organs having a high affinity to adjacent fat tissue (Odening 1961; Odening 1963; Hiepe 1985). In these paratenic hosts, larval trematodes do not develop any further but can survive several host changes without any loss of infectivity (Odening 1961; Hiepe 1985). After ingestion of an AM infected paratenic or second intermediate host by a definitive host, the mesocercariae go through a somatic migration. After passing the lungs, they evolve into metacercariae and later into adult trematodes in the small intestine where they achieve sexual maturity (Odening 1961).

As wild boars are paratenic hosts of *A. alata*, human infection with this trematode through consumption of undercooked or raw wild boar meat or raw meat products cannot be excluded (BfR 2017). Further, *A. alata* has already been classified as zoonotic parasite of risk group 2 by the Federal Office for the Environment (FOEN) and the Federal Office of Public Health (FOPH) in Bern, Switzerland (Gottstein 2013).

The consumption of game meat in Germany has risen over the past decade. According to the German Hunting Association (2019a) which collects data relating to game in Germany, to date, 60% of all Germans consume game meat at least once a year. This amounts to a 25% increase compared to 2009 (German Hunting Association 2019a). The most popular game meat in Germany is wild boar meat and more than 14 tons of wild boar meat were consumed during the hunting season 2018/2019 alone (German Hunting Association 2019a). Traditionally, game meat is consumed fully cooked. However, cooking methods are altering showing a trend towards consumption of inadequately cooked game meat with a pink core as well as raw sausage products (Richomme et al. 2010; Franssen et al. 2017; BfR 2018).

To date, no case reports of human alariosis caused by *A. alata* exist. However, some cases of human infection with *Alaria* spp. respectively *A. americana* mesocercariae have been reported. McDonald et al. (1994) published two cases of intraocular infection with *Alaria* spp. mesocercariae. Both patients probably became infected by consumption of undercooked infested frog legs in local Asian restaurants in California, United States (McDonald et al. 1994). Another case report described a human infection with most likely *Alaria* spp. mesocercariae leading to pulmonary disease and subcutaneous granuloma on the chest (Kramer et al. 1996). The patient was presumably infected through the consumption of raw wild goose meat during a hunting trip in Canada (Kramer et al. 1996). Further, Freeman et al. (1976) reported a serious generalized human infection with *A. americana* after consumption of insufficiently cooked frog legs. The patient developed serious bilateral pneumonia and died of pulmonary bleeding. Also, Odening (1961) demonstrated by experimental infection of a rhesus monkey that primates function as paratenic hosts of *A. alata*. Therefore, clinical disease in humans due to consumption of viable meat or meat products infested with *A. alata* cannot be excluded (Odening 1961).

Article 28 (6) of the Implementing Regulation of the European Commission (EU) 2019/627 (2019) details that carcasses infected with parasites have to be declared unfit for human consumption. Therefore, regionally high *A. alata* prevalences can result in financial losses for hunters and reduce their motivation for hunting (personal communication hunters, expert discussion in October 2016, BfR), increasing e.g. wild boar population densities. This in turn can have detrimental effects on animal disease control and increase crop damage (Massei and Genov 2004).

In 2015, wild boars were tested for the occurrence of *A. alata* in eight German federal states within the framework of the national zoonosis monitoring (BVL 2016). In this study, 4.7% (45/949) of all sampled wild boars tested positive for *A. alata* using the *A. alata* mesocercariae migration technique (AMT) (Riehn et al. 2010). The monitoring also demonstrated regional differences between prevalences in different federal states. For example, the prevalence of Brandenburg was 8.0% (13/163) and therefore almost twice as high as the average prevalence (BVL 2016).

Therefore, the aim of this study was to collect long-term data on the occurrence of *A. alata* in wild boars from Brandenburg to gain a better understanding of temporal and spatial fluctuations in this federal state over time.

## Materials and methods

### Study design

Using the Epitools Epidemiological Calculator by Sergeant (2018), a required sample size of 114 wild boars was calculated with a confidence level of 95% based on the prevalence of 8.0% determined for the German federal state of Brandenburg during the national zoonosis monitoring in 2015 (BVL 2016). Further, the average annual hunting bag of Brandenburg from 2008/09 to 2018/19 (71708 wild boars (German Hunting Association 2019b)) was used as an estimation of the population size.

The sampling was carried out during hunts organized by the German Federal Forest Service, the Frankenförder Forschungsgesellschaft mbH, and the German Institute for Federal Real Estate (BImA). All animals were legally hunted for human consumption and access to post-mortem sampling was kindly provided by the German Federal Forest Service.

Animal age was defined by the hunters based on teeth eruption, teeth replacement, and physical appearance (Habermehl 1961; Bier et al. 2020). Further, wild boars were divided in three age groups (0, 1, 2). Wild boars younger than 1 year belong to age group 0 and 1 year old animals are part of age group 1. All wild boars aged 2 years or older belong to age group 2 (Game Management Directive of the German federal states of Brandenburg and Mecklenburg-Western Pomerania 2001; Portier et al. 2014; Bier et al. 2020).

### Laboratory examinations

After each hunt, the whole tongue and about 30 g of abdominal fat tissue were taken from all sampled wild boars, refrigerated at 4 °C and investigated as a pooled sample with the *A. alata* mesocercariae migration technique (AMT) (Riehn et al. 2010) within a maximum of 7 days after the hunt. Using a stereomicroscope, vital AM were identified by their characteristic morphology and movement and quantified. Further, the parasite load per sample was determined (Table 1).

**Table 1:** Prevalence of *Alaria alata* in wild boars in total and by counties, hunting season, sex, and age group

Variable	Category	No. positive/no. tested animals	Prevalence in % (95% CI)	Range of parasite load (mean parasite load)	*p*-value	PR (95% CI)
Total		100/354	28.25 (23.26–33.25)	0–908 (9.60)		
County	Dahme-Spreewald	29/66	43.94 (31.74–56.70)	0–149 (8.12)		
	Havelland	0/16	0.00 (0.00–20.59)	0		
	Märkisch-Oderland	3/26	11.54 (2.45–30.15)	0–4 (0.23)		
	Oberhavel	13/89	14.61 (8.01–23.68)	0–29 (1.25)		
	Oder-Spree	9/44	20.45 (9.80–35.30)	0–53 (1.98)		
	Ostprignitz-Ruppin	15/53	28.30 (16.79–42.35)	0–67 (2.68)		
	Teltow-Fläming	5/15	33.33 (11.82–61.62)	0–9 (1.07)		
	Uckermark	25/39	64.10 (47.18–78.80)	0–908 (62.18)		
Hunting season	2017/2018	29/111	26.13 (18.25–35.32)		0.824	Reference value
	2018/2019	16/53	30.19 (18.34–44.34)			1.155 (0.690–1.934)
	2019/2020	55/190	28.95 (22.61–35.95)			1.108 (0.755–1.627)
Sex	Male	39/145	26.90 (19.88–34.89)		0.564	Reference value
	Female	56/188	29.79 (23.35–36.87)			1.107 (0.783–1.566)
Age group	0	29/148	19.59 (13.53–26.91)		0.001	Reference value
	1	41/130	31.54 (23.67–40.27)			1.610 (1.065–2.433)
	2	25/55	45.45 (31.97–59.45)			2.320(1.500–3.588)

Note: For statistical analysis, we performed the χ^2^-test according to Pearson. The sex and age group of 21 wild boars as well as the origin (county) of six wild boars were not recorded and could therefore not be used for the analysis

The morphological identification of the larvae was confirmed by an *Alaria* spp. specific PCR (Riehn et al. 2011). All AM were stored in ethanol absolute at −20 °C. DNA extraction of a single larva was performed using the QIAamp DNA Mini Kit (QIAGEN, 51306) following an adapted Quick-Start Protocol. DNA elution was performed in two consecutive steps with 25 μl of distilled water incubated on the column at room temperature for 3 to 5 min before centrifugation at 8000 rpm in each step. Then, a PCR targeting a 303 bp fragment of the *A. alata* genome was performed as described by Riehn et al. (2011).

### Statistical analysis

To assess the effect of sex, age group and hunting season on the prevalence of *A. alata*, the χ^2^-test according to Pearson using the software SPSS v. 26 (SPSS Inc., Chicago, IL, USA) was performed. A value of *p* ≤ 0.05 was considered as statistically significant. Further, prevalence ratios (PRs) produced by a robust Poisson regression were evaluated to estimate the strength of association (Martinez et al. 2017).

Wilson score intervals were used as confidence intervals for the prevalences. The confidence intervals for the prevalence ratios (PRs) were based on the Poisson regression. Both intervals are confidence intervals of 95%.

## Results

In this study, a total of 354 wild boars were sampled during the hunting seasons 2017/2018, 2018/2019 and 2019/2020 in eight counties of the German federal state of Brandenburg. In total, 28.3% (100/354, 95% CI: 23.3–33.3%) of all sampled wild boars in the German federal state of Brandenburg tested positive for *A. alata*. Among all *A. alata* positive wild boars, the number of AM isolated by AMT ranged from zero to 908 AM per sample (pooled tongue and abdominal fat tissue) which resulted in a mean parasite load of 9.60 AM per animal. The mean parasite loads of all AM positive counties reached from 0.23 AM per sample in Märkisch-Oderland to 62.18 AM per sample in Uckermark. AM were detected in wild boars from seven of the eight counties. All samples from county Havelland tested negative for AM (0/16). Prevalences of the seven AM positive counties of Brandenburg ranged from 11.5 (3/26, 95% CI: 2.5–30.1%) in Märkisch-Oderland to 64.1% (25/39, 95% CI: 47.2–78.8%) in Uckermark (Table 1).

In the hunting season 2017/2018, the prevalence was 26.1% (29/111, 95% CI: 18.3–35.3%). In the following hunting season (2018/2019), the prevalence increased to 30.2% (16/53, 95% CI: 18.3–44.3%) and decreased again to 28.9% (55/190, 95% CI: 22.6–36.0%) during the hunting season 2019/2020. Statistically significant differences could not be identified (Table 1; *p* = 0.824).

Further, no association between sex and *A. alata* positivity was determined (Table 1; *p* = 0.564).

However, the prevalence increased with age: 19.6% (29/148, 95% CI: 13.5–26.9%) of all animals of age group 0 were *A. alata* positive. The prevalence rose up to 31.5% (41/130, 95% CI: 23.7–40.3%) in age group 1 and finally to 45.5% (25/55, 95% CI: 32.0–59.5%) in age group 2. The correlation between age and frequency of AM positive wild boars was statistically significant (Table 1; *p* = 0.001). With a PR value of 2.32 (CI: 1.50–3.59; Table 1), wild boars of age group 2 had a prevalence of *A. alata* that was 2.32 times greater than wild boars of age group 0 (Table 1).

## Discussion

Wild boar meat is the most popular game meat in Germany (German Hunting Association 2019a) and can pose a risk of human infection with several foodborne parasites (Ruiz-Fons 2017). Due to a lack of knowledge on recent prevalence data of *A. alata* in German wild boars, the aim of this study was to examine the occurrence of *A. alata* in wild boars in the German federal state of Brandenburg over a longer period of time. The federal state of Brandenburg was chosen for this study because of a comparatively high prevalence (8.0%) of *A. alata* during the zoonosis monitoring in 2015.

In this survey, the average prevalence of *A. alata* in wild boars from Brandenburg between 2017 and 2020 was 28.3% (100/354) (Table 1). Compared to some other European prevalence studies of *A. alata* in wild boars, the total prevalence of our study is clearly higher than those found in France (0.6% or 169/27,582) (Portier et al. 2014), Italy (1.0% or 1/100) (Gazzonis et al. 2018), Czech Republic (6.8% or 15/221) (Paulsen et al. 2013), Austria (6.7% or 30/451) (Paulsen et al. 2012) and northern Serbia (3% or 6/200) (Gavrilović et al. 2019). The average prevalence of 4.7% determined in the German national zoonosis monitoring in 2015 (BVL 2016) falls into the mid-range of prevalences mentioned above.

Interestingly, prior studies conducted in the Eastern parts of Germany all showed relatively high prevalences in comparison to other European countries. However, the *A. alata* prevalence of 28.3% detected in our study was nearly 2.5 times higher than the prevalence of 11.5% demonstrated by Riehn et al. (2012) and even more than 3.5 times higher than the prevalence of 8.0% determined during the national zoonosis monitoring in 2015 (BVL 2016).

Smaller detection rates in other studies might be due to the use of less sensitive methods that were not explicitly developed for AM detection. In the studies from France and northern Serbia for example, all AM were detected by artificial digestion with a magnetic stirrer as used for routine *Trichinella* inspection (Gavrilović et al. 2019). This method is not as sensitive as the AMT described by Riehn et al. (2010) as the mesocercariae can be damaged during digestion (Gavrilović et al. 2019; Portier et al. 2014). According to Riehn et al. (2012), 11.5% (33/286) of all wild boars which tested negative for *A. alata* by artificial digestion were actually AM positive by AMT. Therefore, the true prevalences in the studies in both these countries are presumably higher than determined (Portier et al. 2014). Further, the examination of muscle samples which are not entirely suitable for AM testing could be another reason for smaller detection rates found in other studies. However, in most of the mentioned studies, samples from diaphragm or tongue which both are appropriate tissues (Riehn et al. 2010) were used for AM examination (Riehn et al. 2012; Portier et al. 2014; Gazzonis et al. 2018; Gavrilović et al. 2019). In two studies, mixed samples including diaphragm muscle, glandular and adipose tissue were tested for AM (Paulsen et al. 2012; Paulsen et al. 2013). These sample tissues are also suitable for AM detection due to the affinity of AM to fat, connective and glandular tissue as described by Odening (1961) and Riehn et al. (2010). For this reason, the selection of inappropriate sample material used for AM detection is not an explanation for the lower prevalences found in the studies mentioned above compared to our results.

The current study area, the federal state of Brandenburg, is very rich in lakes, rivers and wetlands (Knittel 2020). This landscape structure offers beneficial conditions for the development and spread of *A. alata* as the parasite’s life cycle is associated with intermediate hosts living in or close to surface waters. This could be an explanation for the relatively high prevalence in Brandenburg compared to some of the studies mentioned above.

To find a potential explanation for the wide variation of the prevalence of 28.3% in our study compared to the prevalence of 11.5% determined by Riehn et al. (2012), weather data of Brandenburg provided by the Climate Data Center were analyzed. We focused on the annual average of both the air temperature and the precipitation level from 2007 to 2009 and from 2015 to 2017, the time frames of the two studies. These were chosen due to the 2-year life cycle of *A. alata*. As reviewed by Möhl et al. (2009), miracidiae maturate for about 1 year in a water snail host before releasing cercariae in the water. Therefore, it should take about 2 years until weather conditions can lead to a noticeable effect on the prevalence of *A. alata* in paratenic hosts such as wild boar.

There were no significant differences in the average temperature during the two studies (10.13 °C vs 9.99 °C) (Climate Data Center 2020a, b).

Between 2007 and 2009, the average annual total precipitation was 663 mm (Climate Data Center 2020c) in comparison to 600 mm between 2015 and 2017 (Climate Data Center 2020d). This variation, however, does not explain the higher prevalence we found in our study indicating that a more in-depth knowledge of both the regional landscape structure as well as the local weather conditions are necessary to understand the size of the parasitic biomass in an area. Further, the higher prevalence in our study compared to the prevalence determined by Riehn et al. (2012) could be justified by an increasing prevalence of finals hosts (e.g. raccoon dogs) in this area. Thus, in the hunting years 2017/18, 2018/19, and 2019/20 which are included in our study, an annual number of 7207, 6572 respectively 6210 raccoon dogs were shot in the federal state of Brandenburg (German Hunting Association 2021). This is a clear rise compared to the hunting years 2009/10 and 2010/11 which were included in the study by Riehn et al. (2012). In these years, an annual number of 5860 respectively 5654 raccoon dogs were shot in Brandenburg (German Hunting Association 2021). Raccoon dogs are known to serve as definitive hosts of *A. alata* (Thiess 2006). Therefore, a growing number of raccoon dogs might be one plausible explanation for the prevalence increase of AM in wild boars in Brandenburg we observed in our survey.

In this study, differences between the prevalences of the eight sampled counties were apparent: While the county Havelland (western part of Brandenburg) had an occurrence of 0.0% (0/16), the prevalence in Dahme-Spreewald (southern part of Brandenburg) was 43.9% (29/66) and in the county Uckermark (northeastern part of Brandenburg) even 64.1% (25/39) (Table 1). These results of a heterogeneous distribution of AM positive wild boars are in line with the findings of the prevalence studies in France (Portier et al. 2014), Austria (Paulsen et al. 2012) and Czech Republic (Paulsen et al. 2013). Due to the small sample size in the county Havelland, the determined occurrence of 0.0% is most probably not representative and needs further verification.

In our survey, a statistically significant correlation between prevalence and age group of the sampled wild boars could be observed. We found a continuous increase of the prevalences of *A. alata* between wild boars of age groups 0 (19.6%), 1 (31.5%) and 2 (45.5%). Similarly, Paulsen et al. (2013) observed a significantly lower prevalence of *A. alata* in wild boars at the age of 1 year and younger (4/22; 18.2%) in comparison to those older than 1 year (11/16; 68.8%). In agreement with Paulsen et al. (2013), it can be assumed that the probability for wild boars to become infected with *A. alata* by eating AM infested prey increases with age.

Similar to the findings in previous studies of Paulsen et al. (2013) and Riehn et al. (2012), a statistically significant association between sex and prevalence of *A. alata* could not be determined.

In conclusion, the regionally very high occurrence of AM in wild boar in Brandenburg, Germany, and the unclear pathogenicity highlight that both further research and discussions on the pathogenicity of the parasite as well as the suitability of cold or heat treatment to kill AM in wild boar meat are needed.

## References

[CR1] BfR (2017) Wildschweinfleisch kann den Duncker´schen Muskelegel enthalten. Aktualisierte Stellungnahme Nr. 011/2017 des BfR vom 27. Juni 2017

[CR2] BfR (2018) Wildfleisch: Gesundheitliche Bewertung von humanpathogenen Parasiten Stellungnahme Nr. 045/2018 des BfR vom 21. Dezember 2018

[CR3] Bier NS, Stollberg K, Mayer-Scholl A, Johne A, Nöckler K, Richter M (2020). Seroprevalence of Toxoplasma gondii in wild boar and deer in Brandenburg, Germany. Zoonoses Public Health.

[CR4] BVL (2016) Zoonosen-Monitoring 2015. Gemeinsamer Bericht des Bundes und der Länder. Berichte zur Lebensmittelsicherheit, BVL-Report 11.2: 30–31

[CR5] Climate Data Center (2020a) Data retrieved from Climate Data Center (CDC): Jahresmittel der Stationsmessungen der Lufttemperatur auf 2 m Höhe in °C; Version:v19.3 & recent; Abgerufen am 03.11.2020

[CR6] Climate Data Center (2020b) Data retrieved from Climate Data Center (CDC): Jahresmittel der Stationsmessungen der Lufttemperatur auf 2 m Höhe in °C; Version:v19.3 & recent; Abgerufen am 21.07.2020

[CR7] Climate Data Center (2020c) Data retrieved from Climate Data Center (CDC): Monatssumme der Stationsmessungen der Niederschlagshöhe in mm; Version:v19.3 & recent; Abgerufen am 04.11.2020

[CR8] Climate Data Center (2020d) Data retrieved from Climate Data Center (CDC): Monatssumme der Stationsmessungen der Niederschlagshöhe in mm; Version:v19.3 & recent; Abgerufen am 20.08.2020

[CR9] Deplazes P, Eckert J, Samson-Himmelstjerna v, Zahner H (2012) Lehrbuch der Parasitologie für die Tiermedizin. 3., überarbeitete Auflage 10.1556/AVet.2012.062 23434846

[CR10] EFSA (2018). The European Union summary report on trends and sources of zoonoses, zoonotic agents and food-borne outbreaks in 2017. EFSA J.

[CR11] Franssen F, Swart A, van der Giessen J, Havelaar A, Takumi K (2017). Parasite to patient: a quantitative risk model for Trichinella spp. in pork and wild boar meat. Int J Food Microbiol.

[CR12] Freeman RS, Stuart PF, Cullen JB, Ritchie AC, Mildon A, Fernandes BJ, Bonin R (1976). Fatal human infection with mesocercariae of the trematode Alaria americana. Am J Trop Med Hyg.

[CR13] Game Management Directive of the German federal states of Brandenburg and Mecklenburg-Western Pomerania (2001) Gemeinsame Richtlinie für die Hege und Bejagung des Schalenwildes der Länder Brandenburg und Mecklenburg-Vorpommern (Wildbewirtschaftungsrichtlinie) vom 24. September 2001 (ABl./01, [Nr. 51], S.859). https://bravors.brandenburg.de/de/verwaltungsvorschriften-216896

[CR14] Gavrilović P, Pavlović I, Todorović I (2019). Alaria alata mesocercariae in domestic pigs and wild boars in South Banat, northern Serbia. Comp Immunol Microbiol Infect Dis.

[CR15] Gazzonis AL, Villa L, Riehn K, Hamedy A, Minazzi S, Olivieri E, Zanzani SA, Manfredi MT (2018). Occurrence of selected zoonotic food-borne parasites and first molecular identification of Alaria alata in wild boars (Sus scrofa) in Italy. Parasitol Res.

[CR16] German Hunting Association (2019a) Deutschland liebt Wildbret vom Wildschwein” Retrieved from https://www.jagdverband.de/deutschland-liebt-wildbret-vom-wildschwein

[CR17] German Hunting Association (2019b) Jahresstrecke Schwarzwild (Wildschweine) 2018/2019. Retrieved from https://www.jagdverband.de/jagd-und-wildunfallstatistik

[CR18] German Hunting Association (2021) Jahresstrecke Marderhund. Data retrieved from https://www.jagdverband.de/sites/default/files/2021-01/2021-01_Infografik_Jahresstrecke_Marderhund_2019_2020.jpg

[CR19] Gottstein B (2013). Einstufung von Organismen. Modul 3: Parasiten. Stand Januar 2013.

[CR20] Habermehl K-H (1961) Altersbestimmung bei Haustieren, Pelztieren und beim jagdbaren Wild: Die Altersbestimmung beim Wildschwein (*Sus scrofa ferus*). Verlag Paul Parey, Berlin und Hamburg, pp. 181–185

[CR21] Hiepe T (1985) Lehrbuch der Parasitologie Bd 3: Veterinärmedizinische Helminthologie. Gustav Fischer Verlag, Stuttgart

[CR22] Knittel T (2020) Gewässer in Brandenburg. Data retrieved from https://www.in-berlin-brandenburg.com/Brandenburg/Urlaub/Gewaesser/

[CR23] Kramer MH, Eberhard ML, Blankenberg TA (1996). Respiratory symptoms and subcutaneous granuloma caused by mesocercariae: a case report. Am J Trop Med Hyg.

[CR24] Martinez BAF, Leotti VB, Silva G d S e, Nunes LN, Machado G, Corbellini LG (2017) Odds ratio or prevalence ratio? An overview of reported statistical methods and appropriateness of interpretations in cross-sectional studies with dichotomous outcomes in veterinary medicine. Front Vet Sci 4(193) 10.3389/fvets.2017.00193 PMC5686058 29177157

[CR25] Massei G, Genov P (2004). The environmental impact of wild boar. Galemys.

[CR26] McDonald HR, Kazacos KR, Schatz H, Johnson RN (1994). Two cases of intraocular infection with Alaria mesocercaria (Trematoda). Am J Ophthalmol.

[CR27] Möhl K, Grosse K, Hamedy A, Wuste T, Kabelitz P, Lucker E (2009). Biology of Alaria spp. and human exposition risk to Alaria mesocercariae—a review. Parasitol Res.

[CR28] Odening K (1961). Der Dunckersche Muskelegel kann experimentell auf Affen übertragen werden. Monatshefte für Veterinärmedizin.

[CR29] Odening K (1963) Zur Diagnostik der Mesocercarie von Alaria alata, eines möglichen Parasiten des Menschen in Europa, an Hand experimenteller Befunde beim Affen. Mber Dtsch Akad Wiss Berlin 5:385–390

[CR30] Paulsen P, Ehebruster J, Irschik I, Lücker E, Riehn K, Winkelmayer R, Smulders FJM (2012). Findings of Alaria alata mesocercariae in wild boars (Sus scrofa) in eastern Austria. Eur J Wildl Res.

[CR31] Paulsen P, Forejtek P, Hutarova Z, Vodnansky M (2013). Alaria alata mesocercariae in wild boar (Sus scrofa, Linnaeus, 1758) in south regions of the Czech Republic. Vet Parasitol.

[CR32] Portier J, Vallée I, Lacour SA, Martin-Schaller R, Ferté H, Durand B (2014). Increasing circulation of Alaria alata mesocercaria in wild boar populations of the Rhine valley, France, 2007-2011. Vet Parasitol.

[CR34] Rentería-Solís Z, Kołodziej-Sobocińska M, Riehn K (2018). Alaria spp. mesocercariae in Eurasian badger (Meles meles) and wild boar (Sus scrofa) from the Białowieża Forest, north-eastern Poland. Parasitol Res.

[CR35] Richomme C, Afonso E, Tolon V, Ducrot C, Halos L, Alliot A, Perret C, Thomas M, Boireau P, Gilot-Fromont E (2010). Seroprevalence and factors associated with Toxoplasma gondii infection in wild boar (Sus scrofa) in a Mediterranean island. Epidemiol Infect.

[CR36] Riehn K, Hamedy A, Grosse K, Zeitler L, Lücker E (2010). A novel detection method for Alaria alata mesocercariae in meat. Parasitol Res.

[CR37] Riehn K, Hamedy A, Alter T, Lucker E (2011). Development of a PCR approach for differentiation of Alaria spp. mesocercariae. Parasitol Res.

[CR38] Riehn K, Hamedy A, Grosse K, Wüste T, Lücker E (2012). Alaria alata in wild boars (Sus scrofa, Linnaeus, 1758) in the eastern parts of Germany. Parasitol Res.

[CR39] Ruiz-Fons F (2017). A review of the current status of relevant zoonotic pathogens in wild swine (S us scrofa) populations: changes modulating the risk of transmission to humans. Transbound Emerg Dis.

[CR40] Sergeant E (2018) Epitools epidemiological calculators. Ausvet. Retrieved from https://epitools.ausvet.com.au/oneproportion

[CR41] Takeuchi-Storm N, Al-Sabi MN, Thamsborg SM, Enemark HL (2015). Alaria alata Mesocercariae among feral cats and badgers, Denmark. Emerg Infect Dis.

[CR42] Thiess A (2006) Untersuchungen zur Helminthenfauna und zum Vorkommen von Trichinella sp. beim Marderhund (Nyctereutes procyonoides) in Brandenburg. FU Berlin. Digitale Dissertation. Retrieved from https://refubium.fu-berlin.de/handle/fub188/11698

